# Implementation of a Quality Improvement Tool “Recover25” to Guide the Care of Patients Experiencing Prolonged Critical Illness: A Mixed-Method Feasibility Study

**DOI:** 10.1097/CCE.0000000000001265

**Published:** 2025-05-13

**Authors:** Laura Allum, Leah Homden, Nicholas Hart, Bronwen Connolly, Natalie Pattison, Louise Rose

**Affiliations:** 1 Florence Nightingale Faculty of Nursing, Midwifery and Palliative Care, King’s College London, James Clerk Maxwell Building, London, United Kingdom.; 2 Lane Fox Clinical Respiratory Physiology Research Centre, St Thomas’ Hospital, Guy’s and St. Thomas’ NHS Foundation Trust, London, United Kingdom.; 3 Intermediate Care Southwark, London, United Kingdom.; 4 Centre for Human and Applied Physiological Sciences, King’s College London, London, United Kingdom.; 5 Wellcome-Wolfson Institute for Experimental Medicine, Queen’s University Belfast, Belfast, United Kingdom.; 6 Department of Physiotherapy, The University of Melbourne, Melbourne, VIC, Australia.; 7 University of Hertfordshire, Hatfield, United Kingdom.; 8 East & North Herts NHS Trust, Stevenage, United Kingdom.; 9 Imperial College London, London, United Kingdom.; 10 Imperial College Health Care NHS Trust, London, United Kingdom.; 11 Florence Nightingale Faculty of Nursing, Midwifery and Palliative Care, King’s College London, London, United Kingdom.; 12 Department of Critical Care and Lane Fox Clinical Respiratory Physiology Research Centre, St Thomas’ Hospital, Guy’s and St. Thomas’ NHS Foundation Trust, London, United Kingdom.

**Keywords:** chronic critical illness, intensive care, prolonged mechanical ventilation, quality improvement

## Abstract

**OBJECTIVES:**

Few quality improvement (QI) tools are specifically designed to manage the care of patients experiencing prolonged critical illness. This risks omissions in care. To determine the implementation feasibility and clinician acceptability of our QI tool “Recover25,” we focused on actionable processes of care for patients with an ICU stay of over 7 days and their families.

**DESIGN:**

Parallel convergent mixed-methods feasibility study conducted between February 2024 and May 2024.

**SETTING:**

A mixed ICU in London, United Kingdom.

**SUBJECTS:**

Patients with an ICU stay of more than 7 days, and the staff who care for them.

**INTERVENTIONS:**

We invited representatives of all ICU professions to a weekly QI round.

**MEASUREMENTS AND MAIN RESULTS:**

We recorded the time completed Recover25, the amount and type of actions generated following Recover25 use (i.e., what new care activities did it prompt), and the number and profession of staff attending each round. We administered the Theoretical Framework of Acceptability (TFA) questionnaire and conducted semi-structured clinician interviews. We calculated means (sds) or interquartile ranges (IQRs) (percentiles) of time to complete and a number of actions generated. We analyzed and integrated qualitative data using framework analysis informed by the TFA. “Recover 25” was used 34 times (65%) of 52 opportunities with 26 patients. Median (IQR) Recover25 completion time was 9.75 minutes (8.2–14.9 min) with a completion rate of 96% (89–100%). Recover25 usage prompted a median of 1 (IQR) (1–2) new action. There was a mean of 4 (sd 2) interprofessional team members attending each QI round. Nineteen clinicians completed 33 TFA questionnaires and 11 interviews. Recover25 was perceived as acceptable, with 94% reporting it aligned with their principles of good care, 85% perceiving it as a coherent intervention, and 67% perceiving it was effective. Interview data showed participants valued the emphasis on person-centered care and highlighted ways to improve implementation.

**CONCLUSION:**

Recover25 was perceived as feasible to implement and acceptable by staff. Further work is needed to understand the effects on patient experience and outcomes.

**KEY POINTS**

**Question:** Is a quality improvement tool, “Recover25” focusing on important actionable processes of care for prolonged ICU stay patients feasible to implement and acceptable to staff?

**Findings:** We found “Recover25” was feasible to implement and perceived as acceptable by the interprofessional team.

**Meaning:** “Recover25,” a quality improvement tool designed for the needs of patients with prolonged critical illness, was acceptable and feasible with future work needed to determine clinical impact, implementation factors, and sustainability.

Patients experiencing a prolonged ICU stay require a change in focus to their care, with different actionable processes of care to those required by the acutely critically unwell. These patients are often awake, maybe weaning from ventilation and/or tracheostomy, and are able to participate in rehabilitation and decisions about their care (1–3). However, they may not be given adequate assistance to communicate their needs (4), require stimulation and orientation to prevent delirium (5) and usually require multilayered ventilator weaning plans (6). There are risks to their prolonged stay: immobility and delirium contribute to increased mortality and long-term dysfunction including pain, difficulties returning to previous physical and social function, and cognitive decline (7, 8). These risks can be moderated somewhat with the prioritization of care activities such as effective nutrition, mobilization and delirium management, but these activities and others have varying implementation in ICU (9, 10)

Quality improvement (QI) tools are used in the ICU to structure and standardize care. They can prevent errors of omission by acting as a cognitive aid promoting best practices and are often used during ward rounds or handovers. Our recent scoping review of QI tools used in the ICU demonstrated that the majority have a positive impact on patient or process of care outcomes (11–14), including reduced mortality, hospital-acquired infections, and delirium. However, very few QI tools are designed with the needs of prolonged-stay patients in mind, and so may not equate to high-value care or meet the complex needs of this population (1, 11, 15). This is a concern given that there are modifiable clinical activities that contribute to the development of prolonged critical illness (9, 16).

Our group previously (1–3, 17) used experience-based co-design methods to develop a QI tool for ICU patients with prolonged stays, the “Recover25” tool. The implementation of this tool might be impacted by common barriers to QI in ICU, such as the instability and acuity of caseload, a complex technical environment (18), and whether staff deem the intervention acceptable and useful (19). Our objectives in this study were, therefore, to determine implementation feasibility and clinician acceptability of the “Recover25” tool.

## METHODS

We used the Template for Intervention Description and Replication (TiDieR) checklist (20) to describe the implementation of Recover25. More information on methods can be found there (**Supplementary Information 1**, https://links.lww.com/CCX/B509).

### Study Design

We conducted a parallel, convergent mixed-methods implementation feasibility study of Recover25 in a general adult ICU (15 beds) in a tertiary-level academic hospital in London, United Kingdom. We selected a mixed-methods design to understand the reasons for quantitative ratings of feasibility and acceptability and to inform future implementation work (21). Both datasets were prioritized equally.

### Participants

All members of the interprofessional team participating in Recover25 rounds were eligible to complete questionnaires and participate in interviews. Patients 18 years and over with an ICU stay of greater than 7 days were eligible for Recover25 completion unless medically unstable or an end-of-life pathway. There is no consensus as to what constitutes a prolonged ICU stay (22–24), and we chose an over 7-day cutoff to reflect the lower boundary of persistent critical illness development identified by Darvall et al (25).

### Sample Size

Our study required two samples: a sample of patients to assess tool feasibility and staff to assess acceptability. Feasibility studies typically recruit a median (interquartile range [IQR]) of 30 participants (20–50 participants) (26, 27). We, therefore, aimed to implement Recover25 on approximately 30 patients. We did not predetermine a sample size for our interviews; instead, we aimed to interview at least one person from each profession attending Recover25 rounds.

### Recover25 Intervention: Planning Implementation Strategy

Before using Recover25, we sought staff stakeholder opinion on tool format and implementation (Supplementary Information 1, https://links.lww.com/CCX/B509). The conclusion from this was to conduct the weekly round at the bedside of eligible patients, using Recover25 (final version in **Supplementary Information 2**, https://links.lww.com/CCX/B509) as a prompt for questions to ask the patient or family member, filled in at the bedside by any member of staff with the support of all members of the interprofessional team involved in their care. We implemented Recover25 for a 12-week period (February 2024 to May 2024). Email reminders were sent the day before each Recover25 round. Informational posters outlining the study were displayed in the ICU and relatives’ rooms to raise awareness among patients and family members.

### Data Collection

We collected baseline demographic and clinical data on patients, including age, sex, ICU day, diagnostic specialty, current organ support, and ventilator status, to understand generalizability of participants. We identified eligible patients in the ICU when a Recover25 round occurred, Recover25 completion rates (in full or part completion), completion time, and which/how many interprofessional team members attended the round. We recorded what actions resulted from Recover25 (e.g., referrals to other professions or implementing patient-centered sleep strategies).

Staff attending each Recover25 round were asked to complete the seven-item Theoretical Framework of Acceptability (TFA) questionnaire anonymously. This questionnaire has not yet been validated but has been widely adopted for a range of healthcare interventions (21, 28), including for staff in the ICU (29, 30). It asks participants to score seven constructs (affective attitude, burden, perceived effectiveness, ethicality, intervention coherence, opportunity costs, and self-efficacy) scored on 1–5 Likert scales (**Supplementary Information 3**, https://links.lww.com/CCX/B509). We conducted semi-structured interviews (**Supplementary Information 4**, https://links.lww.com/CCX/B509) exploring Recover25 acceptability, the impact of Recover25 on patients and staff, and barriers to implementation. Further information can be found in **Supplementary Information 5** (https://links.lww.com/CCX/B509).

### Ethical Considerations

We obtained research ethics approval from the London-Southeast Research Ethics Committee (reference 19/LO/0328, Actionable Processes of Care for Persistent Critical Illness, Approved September 7, 2023), and we followed procedures in accordance with their ethical standards and with the Helsinki Declaration of 1975. An opt-out process was approved for patients. Written informed consent was obtained from staff participating in interviews using a signed participant information sheet, with approval to audio record using a dictaphone or videophone software. Implied consent was indicated by the TFA questionnaire return.

### Analysis

#### Qualitative

Two members of the team (L.A., L.H.) independently analyzed the interview data employing framework analysis. The framework method allowed us to combine deductive and inductive approaches (31). We initially coded the interviews inductively to look for descriptions of staff experiences using the tool. The TFA was then used to structure these codes, with any codes that were not part of the TFA (such as suggestions for future implementation of Recover25) retained.

#### Quantitative

We determined counts and proportions for tool completion rates (entire/each item), and after checking for normality of distribution, we calculated means (sd) or medians (IQR) for Recover25 completion rates and the number of items actioned. We used descriptive statistics for patient demographic characteristics. We scored the TFA questionnaire items as recommended (21).

#### Integration

We used merging integration (32) with each TFA construct having an overall quantitative rating, indicating, for example, how ethical participants deemed Recover25 to be, and qualitative data illustrating what informed this rating; for example, the intervention was deemed ethical because it was patient-centered. We did not set a definition of feasibility or acceptability before the study started. Instead, we used the qualitative findings to contextualize the quantitative ratings and inform whether and how to change implementation processes to improve feasibility and acceptance in future work. We used the Expert Recommendations for Implementing Change (ERIC) framework (33) to structure our findings of pertinent factors for further implementation.

## RESULTS

During the 12-week data collection, Recover25 was used on 34 of 52 opportunities (65%) for 26 eligible patients. Of the 26 patients, 22 (85%) were male, mean (sd) age of 60 years (13.9 yr), and median (IQR) ICU stay of 12.5 days (29.5–11 d) (**Table 1**). The most common reasons for not using Recover25 included insufficient staff present with knowledge of the patient and time pressures for staff (**Table 2**).

**TABLE 1.** Demographic and Clinical Characteristics

Admission Data, *n* (%) Unless Otherwise Stated (*n* = 26)	
Age, median (IQR)	61 (50.5–74)
Sex	
Male	22 (85)
Ethnicity	
Not disclosed	6 (23)
White British	13 (50)
White—any other	4 (16)
Asian/Asian British	2 (8)
Black British	1 (4)
Previous level of mobility	
Fully independent	21 (81)
Walks with an aid or assistance	5 (19)
Previous level of function	
Fully independent	21 (81)
Requires assistance	5 (20)
Admission reason	
Medical	23 (88)
Elective surgical	3 (12)
Emergency surgical	1 (4)
**Day 7 Clinical Information (please note: One Patient Readmitted, Therefore, *n* = 27)**	
Airway	
Tracheostomy	5 (19)
Endotracheal tube	4 (15)
Own	18 (67)
Ventilation status	
Ventilated	6 (22)
Noninvasive ventilation	2 (7)
Unventilated	19 (70)
Renal replacement therapy during admission	
No	16 (59)
Yes	11 (41)
Vasopressors during admission	
No	8 (30)
Yes	19 (70)
Number of invasive lines or tubes	
0–2	13 (48)
3–4	11 (41)
5+	3 (11)
Wounds requiring specialist management^a^	
No	24 (88)
Yes	3 (11)
Total parenteral nutrition	
No	23 (85)
Yes	4 (15)
Sequential Organ Failure Assessment score at admission	6.1 (sd = 2.5)
**Discharge Data**	
Length of stay, median (IQR)	12.5 (11–29.5)
Survived ICU	
Yes	22 (81)
No	5 (19)

IQR = interquartile range.

a For example, input from wound specialist, open abdomen.

b Skewed distribution.

**TABLE 2.** Reasons for Not Using Recover25 With Eligible Patients

Reasons for Recover25 Not Being Used	No. of Occasions (%), *n* = 18
Patient awaiting stepdown to ward	3 (14)
Staffing-related, e.g., insufficient team members able to attend or could stay for the quality improvement round	11 (50)
Tool-related, e.g., not enough time to use with all eligible patients that day	4 (18)

Recover25 completion took a median (IQR) 9.8 minutes (8.2–14.9 min) per patient, with all 25 items completed on 16 of 34 occasions (47%) and a median (IQR) item completion rate of 96% (89–100%). There was a mean (sd) of 4 (2) interprofessional team members attending each Recover25 round, with a senior nurse at every round. Rounds occurred at or away from the bedside due to attending staff preference, infection control precautions, or clinical procedures. Recover25 use prompted a median of one new (IQR, 1–2) action, including referring to psychology, initiating a patient diary, and establishing patient-centered sleep strategies (**Table 3**). Further information on Recover25 completion and staff attendance is presented in the TiDieR checklist (Supplementary Information 1, https://links.lww.com/CCX/B509).

**TABLE 3.** Actions Generated by Recover25 Use

Actions	*n* (%)^a^
None	6 (18)
Patient diary started or continued	16 (47)
Referral to psychology	7 (21)
Sleep strategies implemented	7 (21)
Food/nutrition review	3 (9)
Activities to prevent boredom	3 (9)
Rehabilitation strategies review	3 (9)
Other^b^	1 each (3)

a Number of times an action occurred over 34 episodes of use.

b Other = cognitive assessment, delirium assessment, saliva management, trip off unit, refer to occupational therapy, refer to palliative care, tracheostomy change, investigate mood, and whiteboard.

We conducted 11 semi-structured interviews with staff participants who were mostly female (8, 73%) with ICU experience ranging from younger than 2 years to older than 20 years. We received 33 completed TFA questionnaires from the 19 healthcare professionals who attended the rounds, representing a range of professions, including senior nurses, intensivists, pharmacists, and Allied Health professionals (**Supplementary Information 6**, https://links.lww.com/CCX/B509). We summarized and integrated quantitative and qualitative data using a mixed-methods matrix (**Table 4**) and reported data below using the seven TFA domains.

**TABLE 4.** Mixed-Methods Matrix

Theoretical Framework of Acceptability Construct	Subconstruct From Interview Data	*n*/33 (%) of Questionnaire Respondees Who Agreed or Strongly Agreed With Each Characterization of Recover25
Ethicality—the extent to which the intervention has good fit with an individual’s value system	Inclusion of patient	31 (94)
	Recover25structure	
	Interdisciplinary boundaries	
Burden—the perceived amount of effort that is required to participate in the intervention	Time	21 (64)
	Patient’s ability to participate	
	Team familiarity with patient	
	Wording of Recover25	
	Recover25 structure	
Perceived effectiveness—the extent to which the intervention is perceived as likely to achieve its purpose	Is effective	22 (67)
	Is not effective	
Affective attitude—how an individual feels about the intervention	Patient-centeredness	28 (85)
	Family inclusion	
	Comprehensiveness	
Intervention coherence—the extent to which the participant understands the intervention and how it works	Systematic approach	28 (85)
	interdisciplinary team communication	
	Continuity	
	Picking the right patient	
Self-efficacy—the participant’s confidence that they can perform the behaviors required to participate in the intervention	Experience with Recover25	24 (73)
Opportunity costs—the extent to which benefits, profits or values must be given up to engage in the intervention	Competing priorities	14 (42)

### Ethicality

Almost all participants rated Recover25 highly from an ethical perspective, with 31 of 33 (94%) agreeing or strongly agreeing that it was an ethical intervention. Interview data indicated that participants valued how Recover25 placed the patient at the center of care discussions, enhancing their autonomy:

An opportunity for patients to identify their needs and [be] asked for their opinion, it makes them an active participant in their care, which is wonderful—Interviewee 3

A minority of participants identified aspects that they deemed unethical, including the feeling that some items overstepped professional boundaries, particularly for nursing-related items. While most found the Recover25 tick-box sections easy to use, others felt strongly that this was an inappropriate format, preferring free-text formats to encourage open conversation.

### Burden

TFA questionnaire responses indicated 21 of 33 (64%) expressed agreement or strong agreement that the burden presented by Recover25 was acceptable. Mostly, this burden related to the time required to complete Recover25, but this was deemed worthwhile because meeting the interdisciplinary team and patient reduced the need to communicate with each member individually.

Realistically it was an hour that was out of my day for a few patients. That wasn’t loads of time—Interviewee 7

Some participants felt the time commitment was prohibitive, and favored one staff member going to each patient’s bedside to discuss Recover25 items, before sharing findings as an interprofessional team to reduce the time required. This was also proposed due to a perceived awkwardness in asking questions that the patient or family might be less willing to answer in front of a large group, such as questions about their mood.

Several participants spoke of their frustration using Recover25 when patients were not able to input. Some proposed only using Recover25 with a communicative patient and/or family member.

It’s really important to have people that know the patients there or else it’s long and it feels a little bit pointless—Interviewee 1

Although some participants felt the tick-box structure was insufficiently patient-friendly, the structure was easier and quicker, so it reduced the Recover25 burden.

### Perceived Effectiveness

Of the 33 questionnaire responses, 22 (67%) agreed or strongly agreed that Recover25 was likely to be effective in influencing patient care. The reasons include the structured approach it provided, and the benefit of a specific time to discuss long-stay patients as an interprofessional group.

Just by having those 10–15 minutes chatting to the patient and the other clinicians…allowing all of us to kind of add our 2 cents. It probably gives me a bit more of a global picture—Interviewee 4

However, three of 33 (9%) questionnaire responses indicated Recover25 was ineffective, adding insufficient information to justify an extra meeting outside of normal rounds. Participants also expressed concern that there was no accountability for actions identified by Recover25. Lack of interprofessional representation on rounds, attributed to competing demands on time, was considered the primary barrier to Recover25 effectiveness.

The most meaningful discussions are when we have representation from all of our AHPs… but if some of those people can’t be there… it really erodes the richness of the conversation—Interviewee 5

Attendance by the bedside nursing staff was viewed as integral; however, the Recover25 round often coincided with nursing breaks. Participants suggested that their attendance be facilitated by morning conversations with senior nurses overseeing the ICU to improve break coordination.

Participants felt reporting the impact of Recover25 to the wider ICU team might improve attendance, and advocated for it to be included on the hospital’s electronic medical record as a prompt and for ease of reporting.

### Affective Attitude

Of the 33 questionnaire responses, 28 (85%) agreed or strongly agreed that they liked Recover25 as it reflected their principles of good care. Many participants liked that patients were involved in conversations taking place at the bedside, sometimes expressing surprise at what the patient had articulated. Participants liked that the Recover25 round was conducted during visiting times, therefore, including family in conversations:

Whilst it was sort of around visiting time, that visibility I think is probably really helpful for [families], a chance to be included and to know that we were being quite holistic—Interviewee 2

Participants appreciated the forum to discuss long-stay patients in detail, which was not otherwise occurring. Most participants liked Recover25’s comprehensive nature, feeling reassured that it prevents missing important aspects of care.

### Intervention Coherence

Of the 33 questionnaire responses, 28 (85%) said they agreed or strongly agreed they could understand the mechanism by which Recover25 worked, that is, by establishing a systematic approach to long-stay patients that would prevent care omissions or delays:

You probably would have got there over a week or two with things like whiteboards, sleep masks, et cetera...But it just prompts you to think about those things slightly earlier—Interviewee 7

This was perceived as particularly helpful for less experienced staff, for whom Recover25 prompted investigation of issues not associated with their role:

There have been some things that have come out of it that I wouldn’t have picked up on before. If someone’s got issues with their mood and then you ask them as one of the prompts on the tool…—Interviewee 4

Participants reported that rounding with Recover25 enhanced professional communication, and felt Recover25 helped establish continuity for long-stay patients, often difficult with rotating staff and multiple interprofessional teams:

We do have people in ICU forever and... everyone else rotates, so having some kind of continuity is really important. And that’s what this tool is really good at—Interviewee 6

Several participants suggested changing the inclusion criteria, considering 7 days not representative of a long stay.

### Self-Efficacy

Of the 33 questionnaire responses, 24 (73%) agreed or strongly agreed they felt confident using Recover25. Some participants reported initial difficulties using Recover25 in a conversational way with the patient. However, once familiarized with Recover25, it became easier and quicker to use:

Once you become more familiar with the form, you can sort of whip through it a bit more quickly—Interviewee 2

### Opportunity Costs

Of the 33 questionnaire respondents, 19 (58%) agreed or strongly agreed that using Recover25 interfered with other priorities. Qualitative data suggested several factors including Recover25 completion time, staffing pressures, and the belief that some content was discussed in other meetings. Participants spoke of difficulties managing conflicting demands on their time, and challenges of finding a time that suited most members of the interprofessional team. It was important the meeting had clear benefit and sufficient interprofessional team members in attendance:

I’m ridiculously busy...but I’m prioritizing this because I think it’s gonna have great value. I can really see the potential of it and...I really want to be there and pull in as an MDT—Interviewee 1

While our qualitative and quantitative data indicate convergence in findings, qualitative data provides important insights into implementation barriers and possible solutions. To structure these implementation findings, we used the ERIC framework (25), an expert-informed compilation of Implementation strategies. **Supplementary Information 7** (https://links.lww.com/CCX/B509) shows a plan for further implementation using ERIC, incorporating findings from both this feasibility study and our previous scoping review of ICU QI tools and their implementation (7).

## DISCUSSION

This mixed-methods study explored implementation feasibility of a QI tool, “Recover25,” for patients with an ICU admission over 7 days. It was found feasible; it was used on 65% of eligible patients over a 12-week period, taking less than 10 minutes per patient and with a median 96% item completion rate. Interview data suggest that Recover25 was feasible because it was easy for staff to complete and they deemed it worthwhile for the time taken. Staff described Recover25 as acceptable, with 85% liking and feeling it was a coherent intervention, and particularly because they deemed it an ethical intervention (94%) aligning with their values of good care for patients with prolonged critical illness. Staff told us they felt Recover25 was effective for identifying care omissions or new patient needs. However, concerns about staff buy-in and pressures on staff time were important barriers to effectiveness, particularly since representation from the full interprofessional team was integral to a meaningful discussion. Cultures that foster interprofessional communication have been shown to be associated with lower rates of persistent critical illness development (9).

Participants particularly valued that Recover25 enhanced patient and family-centered care, especially when used at the bedside of an awake patient, or with an advocating family member present. However, some participants felt Recover25 should be used away from the bedside, contrary to evidence suggesting positive outcomes resulting from interprofessional bedside rounds (34–36) including reduced need for separate family meetings (37), and the consequences of poor information provision to patients and families (38, 39). Preference for discussions away from the family and patient may reflect concerns about involving family members in care (40) including the impact on workload, particularly for nurses (41, 42).

We did not set a definition of feasibility and acceptability pre-intervention. Comparable data from our previous scoping review (11) indicates Recover25 has favorable item completion rates (43, 44), enrollment (45), and completion times (46, 47). Our acceptability ratings compare favorably with those of other health interventions assessed using the TFA (48, 49). Adopting changes suggested by participants may improve acceptability and feasibility of Recover25, but this requires further evaluation, likely requiring several steps in the plan, do, study, act (PDSA) model (50) to assess impact. Should this adoption prove successful, next steps should include using the progression criteria model of “Stop, Change, Go” proposed for feasibility studies (27, 51) to determine whether a future randomized controlled trial is worthwhile.

Suggestions for ongoing implementation included incorporating Recover25 in electronic medical records, allowing greater visibility for those unable to attend Recover25 rounds and facilitating documentation of actions and impact initiated by Recover25. Observable impact is a key learning from large-scale ICU QI implementation projects such as The ICU Liberation Collaborative and Keystone ICU Project (52). Participants felt that interdisciplinary engagement would be improved by reporting patient and family feedback on Recover25, as described in our previous scoping review of ICU tools (11).

Approximately one third of opportunities to use Recover25 were missed. Most typically this was due to insufficient staff attendance, often because staff had to leave during the round due to time pressures. Participants questioned the need to include all patients with an ICU stay over 7 days, and we intend to conduct further investigation to understand which patients benefit most from this intervention, including whether there are clinical factors which are associated with benefit. A more targeted inclusion criteria might reduce the burden on staff attending and therefore improve Recover25 delivery.

We used well-integrated mixed-methods analysis in this study, allowing us to understand the factors behind Recover25 implementation feasibility and acceptability, with detailed exploration of implementation barriers and facilitators using a validated implementation science framework. Nine of 11 interviews were conducted by a researcher uninvolved in the development of the Recover25 (L.H.) to reduce response bias. We acknowledge limitations, including that conducting in a single-center limits generalizability and that our patient participants were mostly unventilated via their own airway. We sought feedback only from interprofessional team members attending Recover25 rounds, so have no data on why people did not attend and why some attendees declined to be interviewed. We also did not collect the patient and family member perspective. Crucially, we struggled to establish involvement from nurses in both Recover25 usage and analysis—only three nurses were interviewed, and none were bedside nurses. We recognize that this is a significant underrepresentation of those who provide much of the activities recommended in Recover25, and future work must engage the nursing perspective more successfully to faithfully represent implementation barriers and facilitators.

## CONCLUSIONS

We demonstrated our QI tool “Recover25,” aimed at structuring and standardizing care of patients with prolonged critical illness, was feasible and acceptable to clinical staff. Future research work should employ a PDSA model to explore implementation factors that enhance Recover25 uptake. We will also need to identify appropriate outcome measures pertinent to this impact before an effectiveness-implementation hybrid trial to determine the clinical effect in context, sustainability, and scalability. We advise that policy considers specific QI indicators for this patient population given their specific needs and the consequences of low-value care on them, their family, and the wider health and social care system.

## Supplementary Material
