# Supplementary material for: Implementation of a Quality Improvement Tool “Recover25” to Guide the Care of Patients Experiencing Prolonged Critical Illness: A Mixed-Method Feasibility Study
Source: Crit Care Explor. 2025 May 13;7(5):e1265. doi: 10.1097/CCE.0000000000001265 (PMC12077562; doi:10.1097/CCE.0000000000001265)
Supplement: Supplementary file 1 [file cc9-7-e1265-s001.pdf]

Supplementary Information 1 – TiDieR checklist

**Why:**	A quality improvement tool used for prolonged stay patients in the ICU will prevent omissions and standardise care, 'Recover25'
**What (material):**	A quality improvement tool, largely in checklist form but with opportunities for free text to detail how each process of care was actioned
**What (procedures):**	Recover25 was used once a week for all patients with an ICU stay of more than 7 days unless being palliated or unstable (i.e. blood pressure unable to be maintained on no or low vasopressor support and not on continuous sedation). All interdisciplinary team members were invited to a specific Recover25 round, hoping that all professions would be represented on the QI round, and the QI round would proceed unless no attendees knew the patients involved. We planned for the round to be conducted at the patient's bedside.
**Who provided:**	Recover25 was used for 12 weeks, so some staff members attended frequently and built experience using the tool, and some attendees attended less often or once.
**How (mode of delivery; individual or group):**	A staff member would discuss all points on Recover25 in turn, at the patient's bedside, involving the patient as able and including any family members present, with the support of all members of the interprofessional team involved in their care.
**Where:**	A printout of Recover25 when it was used at the patient's bedside.
**When and how much:**	Once a week for twelve weeks, with all eligible patients.
**Tailoring:**	We sought stakeholder feedback on preferences on format (checklist versus free text) and frequency (weekly versus more often) from stakeholder groups representing the ICU interprofessional team. We sought opinions on when to use Recover25 – e.g. during ICU rounds or starting a new round for prolonged stay patients using Recover25 . We held two meetings with senior nursing staff, two meetings with nursing leadership and education, two interprofessional meetings focusing on clinical governance and oversight, and various emails and informal discussions held with allied health professionals.<br>It was planned that staff would adapt Recover25 questions dependent on a) whether they were relevant to each patient (e.g. for unventilated patients, questions about ventilator weaning should not be asked) b) whether the patient was able to take part and communicate, with the assumption that speaking valves or other communication aids would be used for this purpose c) family were involved if present and d) all staff members were able to contribute to each question with any relevant information.
**Modification:**	Some staff felt there were questions that should not be asked of the patient, such as referrals to palliative care and ICU pharmacotherapy de-escalation. Therefore, depending on which staff members were attending the QI round, sometimes these questions were discussed away from the bedside. In addition, some staff members had strong preferences for conducting the QI rounds at the patient's bedside versus away from the bedside, particularly if the patient was not able to take part.
**How well (planned):**	Recover25 used on all eligible patients each week, with round taking place at patients' bedsides. Recover25 should be completed in full for all eligible patients, except for not applicable items. All members of the interdisciplinary team should be present at each round, with patient and family assisted to take part where possible.
**How well (actual):**	Out of 52 opportunities to use Recover25with 26 patients, it was used 34 times. Reasons for not using Recover25included staffing-related (i.e. insufficient members of the MDT attended or could attend for long enough to use with all eligible patients each week) = 11 or tool-related (i.e. Recover25took too long to use with previous patients, so not enough time to use with all eligible patients that day) = 4. Recover25 had all 25 items completed 16/34 times. On 9/34 occasions, 23 or 24/34 items were completed. 20,21 or 22 items were completed on 5/34 occasions and on 4/34 occasions less than 20 of 25 items were completed. A senior nurse was present for all 12 QI rounds. Occupational therapy and physiotherapy were present for 7 QI rounds each, intensivists were present for 5, psychology for 3, family liaison nurses for 2 and other professions once each (matron (nursing leadership), speech and language therapy, dietitian and physicians' assistant). For 18 patients, Recover25 was used once, for six patients twice, three patients three times, one patient four times and two patients eight times.

Supplementary Information 2 – Recover25 - Prolonged Critical Illness Quality Improvement Tool

Prolonged ICU stay care plan

Date of Admission:

TOPIC	ITEM	NOT DONE (please give a reason)	IN PROGRESS (outline actions)	DONE (outline actions)
1. INVOLVING PATIENT AND FAMILY IN GOAL SETTING AND DECISION MAKING	a. Involve patient in bedside rounds/goals of care discussions as early as able			
	b. Provide regular proactive family meetings to set goals, devise a care plan, and share information. Last family update: ___________			
	c. Identify legal shared decision maker & ensure review of any patient wishes and preferences, advance statements and/or advance decisions to refuse treatment			
2. PROVIDE AIDS TO ENABLE PATIENT TO COMMUNICATE (INCLUDE METHOD TO ACCESS HELP)				
3. PROMOTE PHYSICAL COMFORT AND MINIMIZE COMPLICATIONS	a. Prevent/treat complications associated with prolonged bed rest/ICU stay (i.e., pressure ulcers, constipation, DVTs)			
	b. Provide regular oral care including toothbrushing			
	c. Provide fundamental hygiene and elimination care			
	d. Assess/manage symptoms (pain, breathlessness, tiredness, thirst)			
	e. Ensure adequate nutritional support			
4. PROMOTE SELF-CARE AND RESTORE NORMALCY	a. Enable access to activities (radio, tv, ipad) and personal possessions (including clothes) to prevent boredom, delirium, loneliness, and restore normality - might require a referral to Occupational Therapy			
	b. Use patient diary to aid communication with and provide psychological support for patient and family			

5. OPTIMIZE VENTILATOR WEANING	a. Track and guide ventilator weaning using a structured tool (protocol/individualized weaning plan)			
	b. Assess causes of weaning failure, including respiratory muscle weakness (provide resp muscle training as appropriate) and endocrine function			
	c. Assess readiness to deflate the tracheostomy cuff, downsize or decannulate the tracheostomy as part of the weaning process			
	d. Use strategies to manage excess secretions/inability to cough up secretions			
6. OPTIMIZE PHYSICAL RECOVERY	a. Regular physical rehabilitation (including early mobilisation) with setting and assessment of progress on weekly rehabilitation goals)			
	b. Assess and treat non-respiratory muscle wasting			
7. ASSESS SWALLOWING FUNCTION AND ESTABLISH SAFE RETURN TO NORMAL DRINKING AND EATING<br>- might require a referral to Speech and Language Therapy				
8. DE-ESCALATE (INCLUDING CHANGE TO ORAL INSTEAD OF IV DRUGS) OR STOP ICU PHARMACOTHERAPY AND RESTART PREVIOUS COMORBIDITY PHARMACOTHERAPY)				
9. ASSESS AND TREAT PSYCHOLOGICAL ISSUES INCLUDING ANXIETY, DEPRESSION, AND ACUTE STRESS<br>- might require a referral to Psychology				
10. MINIMIZE DELIRIUM RISK	a. Use patient preferences for strategies to promote sleep, including reducing night-time light/noise			
	b. Review ongoing need for sedation and decrease as able			
11. ENSURE CONTINUITY IN CARE	a. Conduct interprofessional team meetings to discuss patient- and family-centred care plan and setting interprofessional goals			
	b. Appropriate and timely discharge planning (discuss and arrange safe transitions in care location)			
	c. Enable appropriate and timely referral to palliative care			

Supplementary Information 3 – TFA questionnaire adapted from Sekhon (2022).

Please think about the Prolonged ICU Stay Tool and rate the following statements using the scale that follows.

Did you like or dislike the Prolonged ICU Stay Tool?

Strongly dislike	Dislike	No opinion	Like	Strongly like
1	**2**	**3**	**4**	**5**

How much effort did it take to engage with the Prolonged ICU Stay Tool?

No effort at all	A little effort	No opinion	A lot of effort	Huge effort
1	**2**	**3**	**4**	**5**

The Prolonged ICU Stay Tool has improved the management of prolonged ICU stay patients in this ICU.

Strongly disagree	Disagree	No opinion	Agree	Strongly agree
1	**2**	**3**	**4**	**5**

It is clear to me how the Prolonged ICU Stay Tool will help manage the care of prolonged stay ICU patients.

Strongly disagree	Disagree	No opinion	Agree	Strongly agree
1	**2**	**3**	**4**	**5**

How confident did you feel about engaging with the Prolonged ICU Stay Tool?

Very unconfident	Unconfident	No opinion	Confident	Very confident
1	**2**	**3**	**4**	**5**

Engaging with the Prolonged ICU Stay Tool interfered with my other priorities.

Strongly disagree	Disagree	No opinion	Agree	Strongly agree
1	**2**	**3**	**4**	**5**

How acceptable was the Prolonged ICU Stay Tool to you?

Completely unacceptable	Unacceptable	No opinion	Acceptable	Completely acceptable
1	**2**	**3**	**4**	**5**

Supplementary Information 4 – Interview Guide

King's College London

Guy's and St Thomas' NHS Foundation Trust

Patient Identifier **Date**

1. What is your profession in ICU?
2. Can you tell me how long you have been working in ICU?
3. Tell me about how you found using the Prolonged ICU Stay Bedside Care Tool?
4. What do you like about the tool? (what worked well and why?)
5. What do you not like about the tool? (what worked not so well and why?)

   Prompt: Is there anything you would like to change about the tool?

6. How do you think the tool improved the patient/family care?

   Prompt: Does the tool change the way you care? In what way?

   Prompt: And what does care look like for patients and their families without this tool?

7. What barriers did you encounter using the tool?

   Prompt: roughly how long did it take you to use?

8. Is there anything that you think would help make the tool more useful or successful?
9. Anything else?

Supplementary Information 5- Good Reporting of A Mixed Methods Study (GRAMMS)

**(1)** Describe the justification for using a mixed methods approach to the research question	We wanted to understand the the qualitative factors that informed quantitative ratings of acceptability. Quantitative methods were used in part because in the time-pressured ICU not all staff were likely to agree to be interviewed, but would agree to complete a brief survey – therefore more feedback would be provided using mixed methods.
**(2)** Describe the design in terms of the purpose, priority and sequence of methods	Quantitative methods were used to provide an overall score of the elements of acceptability as defined by the TFA for staff. Qualitative methods were used to understand the reasons behind these ratings, to inform future implementation work. Quantitative and qualitative methods were prioritized equally. Participants were asked to complete the TFA survey immediately after each Recover25 round, and results were analysed at the end of the data collection period. Interviews took place over the course of the data collection period and analysed in an ongoing manner.
**(3)** Describe each method in terms of sampling, data collection and analysis	SAMPLING<br>Please see Methods: Sample size<br>DATA COLLECTION<br>Feasibility – Recover25 completion rates (full or part completion), time to complete, which/how many interprofessional team members attended the Recovery25 round. We recorded what actions resulted from using Recover25, such as referrals to other professionals.<br>Acceptability: Each member of staff attending the Recover25 round was asked to complete the TFA when rounds were completed. Each member of staff was invited to a semi-structured interview about their experiences using the tool. Interviews were audio recorded using a Dictaphone or MSTeams and transcribed. Two interviews were conducted by the main researcher (LA), an ICU physiotherapist, and nine by an ICU occupational therapist (LH) uninvolved in the design of Recover25, hoping to encourage honest responses from participants. To enhance rigor, LH listened to recordings of interviews conducted by LA before conducting her interviews, and LA listened to recordings of the first three interviews conducted by LH aiming to provide feedback and standardisation. Field notes were taken by the lead researcher (LA) during each Recover25 round.<br>ANALYSIS<br>Please see Methods: Analysis
**(4)** Describe where integration has occurred, how it has occurred and who has participated in it	Please see Analysis: page 8.
**(5)** Describe any limitation of one method associated with the present of the other method	Results: page 13.
**(6)** Describe any insights gained from mixing or integrating methods	Analysis: page 8. Results: pages 8-13. Discussion: 14-15.

Supplementary information 6 - Interview Participant demographics

Profession	Dietitian	1
	Family Liaison Nurse	1
	Intensivist	1
	Matron (nursing leadership)	1
	Nurse in charge	1
	Occupational Therapist	2
	Physician's Associate	1
	Physiotherapist	2
	Speech and Language Therapist	1
Years Experience	Less than two years	3
	2-5 years	1
	5-10 years	3
	10-20 years	2
	20+ years	2
Sex	Male	3
	Female	8

Supplementary Information 7 - implementation strategies pertinent to further evaluation of Recover25

ERIC strategies applicable to next stage evaluation	
**Change infrastructure**	
Change physical structure and equipment	N/A
Change record systems	
Change service sites	
Start a dissemination organization	
Mandate change [b]	**Likely useful once clinical impact established**
Create or change credentialing and/or licensure standards [b]	N/A
Change liability laws	
Change accreditation or membership requirements	
**Financial strategies**	
Access new funding	N/A
Alter incentive/allowance structures [b]	
Develop disincentives	
Fund and contract for the clinical innovation	
Make billing easier	
Place innovation on fee for service lists/formularies	
Alter patient/consumer fees	
Use capitated payments	
Use other payment schemes	
**Support clinicians**	
Create new clinical teams	N/A
Facilitate relay of clinical data to providers [a, c]	**Might employ**
Revise professional roles	
Remind clinicians [a, b, c]	Continue
Develop resource sharing agreements	N/A
**Provide interactive assistance**	
Facilitation [b, c]	**Employ**
Provide local technical assistance	N/A
Provide clinical supervision [b]	**Employ**
Centralize technical assistance	N/A
**Adapt and tailor to context**	
Use data experts	N/A

Use data warehousing techniques	
Tailor strategies [a, c]	**Employ**
Promote adaptability [a, c]	**Employ**
**Train and educate stakeholders**	
Conduct educational meetings [b, c]	Continue
Conduct educational outreach visits	N/A
Conduct ongoing training [c]	**Employ**
Create a learning collaborative [b]	
Develop educational materials [a, b]	
Distribute educational materials [a, b]	
Provide ongoing consultation [a, b, c]	
Use train-the-trainer strategies [b]	
Make training dynamic	
Shadow other experts [a, c]	Continue
Work with educational institutions	N/A
**Develop stakeholder relationships**	
Build a coalition [a, c]	Continue
Conduct local consensus discussions [c]	
Obtain formal commitments [c]	
Recruit, designate, and train for leadership [b]	**Employ**
Inform local opinion leaders [c]	Continue
Capture and share local knowledge [c]	
Identify and prepare champions [b, c]	
Organize clinician implementation team meetings [b]	**Employ**
Use advisory boards and workgroups [b, c]	
Use an implementation advisor [b, c]	Continue
Promote network weaving	N/A
Model and simulate change [c]	**Employ**
Develop academic partnerships [c]	Continue
Identify early adopters [c]	
Visit other sites [b]	N/A
Develop an implementation glossary	
Involve executive boards	
**Use evaluative and iterative strategies**	
Assess for readiness and identify barriers and facilitators [b, c]	

Conduct local needs assessment [b, c]	Continue
Develop a formal implementation blueprint [c]	**Employ**
Stage implementation scale up	
Audit and provide feedback [a, b, c]	
Conduct cyclical small tests of change	
Develop and implement tools for quality monitoring [a, b, c]	
Develop and organize quality monitoring systems [b, c]	
Purposely reexamine the implementation [c]	
Obtain and use patients/consumers and family feedback [a, b, c]	
**Engage Consumers**	
Involve patients/consumers and family members [b, c]	**Employ**
Prepare patients/consumers to be active participants [b, c]	
Use mass media	N/A
Increase demand	
Intervene with patients/consumers to enhance uptake and adherence [c]	

[a] = suggested by study participants

[b] = identified in our previous scoping review (Allum et al., 2022).

[c] = used in this study
